# Supplementary material for: Nutraceutical Difference between Two Popular Thai Namwa Cultivars Used for Sun Dried Banana Products
Source: Molecules. 2022 Sep 2;27(17):5675. doi: 10.3390/molecules27175675 (PMC9458235; doi:10.3390/molecules27175675)
Supplement: Supplementary file 1 [file molecules-27-05675-s001.zip › molecules-1883000-supplementary.pdf]

Table S1 Correlation matrix of solar dried banana products from Namwa Mali Ong and Nuanchan varieties and the four commercial products

	TPC	Tannin	DPPH	FRAP	Total Phenolic acid	Total Flavoniods
TPC	1					
Tannin	1.000**	1				
DPPH	-1.000**	-1.000**	1			
FRAP	1.000**	1.000**	-1.000**	1		
Phenolic acid	1.000**	1.000**	-1.000**	1.000**	1	
Flavoniods	1.000**	1.000**	-1.000**	1.000**	1.000**	1

**Correlation is significant at the 0.01 level; TPC, Tannin, DPPH, and FRAP by colorimetric analysis; Phenolic acid and Flavonoids by HPLC;

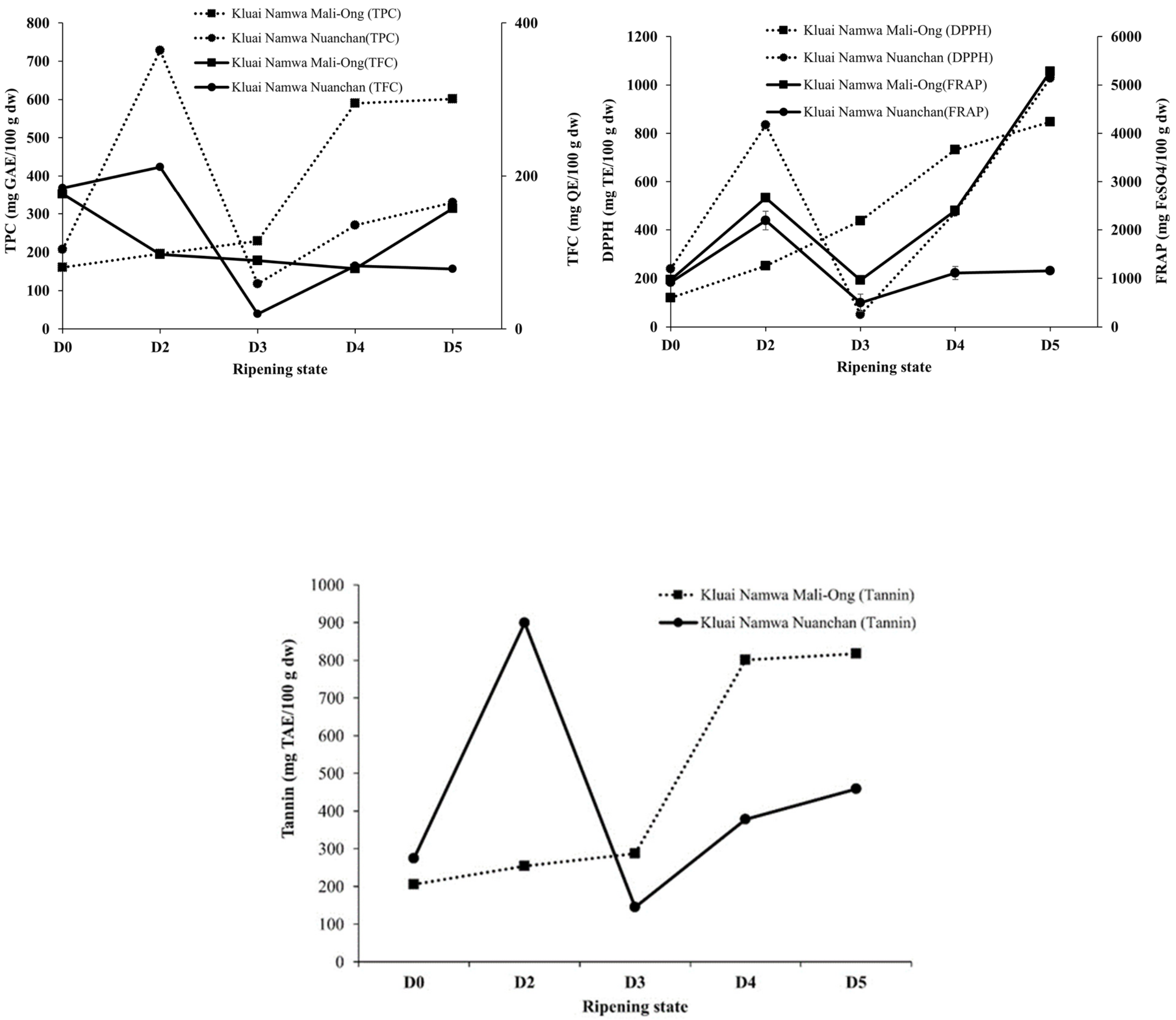

Figure S1 Antioxidant value based on dry mass of Namwa Mali Ong and Nuanchan varieties during ripening stage
